# Prevalence of urinary tract infections in pregnancy in rural Andean communities of Peru

**DOI:** 10.1177/17455057241294215

**Published:** 2024-11-05

**Authors:** Apoorva Venkatesh, Giuliana Sanchez-Samaniego, Daniel Mäusezahl, Jan Hattendorf, Stella Maria Hartinger

**Affiliations:** 1 Swiss Tropical and Public Health Institute, Allschwil, Switzerland; 2 University of Basel, Basel, Switzerland; 3 School of Public Health and Administration, Universidad Peruana Cayetano Heredia, Lima, Peru

**Keywords:** urinary tract infection, pregnancy, screening, active case-finding, prevalence, ALTO, Peru, San Marcos

## Abstract

**Background:**

Urinary tract infections (UTIs) can adversely affect pregnancy, yet their prevalence in rural communities remains poorly understood.

**Objective:**

For an initial evaluation of the UTI burden among pregnant women in the San Marcos province of rural Andean Peru, we aimed to determine the UTI prevalence in the region.

**Design:**

A cross-sectional study was conducted in a subsample of 250 pregnant women enrolled in the Peruvian Andes Multigenerational High Altitude Cohort (ALTO) from 2021 to 2022.

**Methods:**

Structured questionnaires were administered to collect demographic, socioeconomic, maternal health and behavioural data. Urine samples were obtained for dipstick analyses. Descriptive and logistic regression analyses were performed to investigate the association between risk factors and UTI.

**Results:**

The study participants had a median age of 28 years (IQR: 22–33). A UTI prevalence of 27.6% (CI: 22.4%–33.5%) was observed, surpassing estimates from other regions of Peru. Notably, nearly all pregnant women (96%) utilised antenatal care (ANC) services at least once, primarily visiting health centres and community health posts where doctors and obstetricians were the main care providers. In this population, none of the risk factors exhibited statistically significant associations with UTIs.

**Conclusion:**

Our study highlights the prevalence of UTIs among Andean pregnant women in San Marcos and underscores the critical need for routine UTI screening and treatment during ANC visits as recommended by national guidelines. While accessibility to ANC services is not a barrier in this region, enhancing the availability and quality of UTI screening services is crucial.

## Plain language summary

How Common Are Urinary Tract Infections in Pregnant Women in Rural Areas of the Andes in Peru?

Urinary tract infections (UTIs) can cause complications during pregnancy, but we don’t know enough about how common they are in rural areas. This study looked at how many pregnant women in San Marcos, a rural area in the Andes of Peru, have UTIs. The goal was to better understand the problem in this community. We studied 250 pregnant women who were part of a larger research project called the Peruvian Andes Multigenerational High Altitude Cohort (ALTO), conducted between 2021 and 2022. The women answered questions about their health, occupation, education and lifestyle. They also provided urine samples, which were tested for UTIs using a dipstick test. The women in this study were, on average, 28 years old. We found that about 28% of the women had a UTI during pregnancy, which is higher than rates reported in other parts of Peru. Almost all of the women (96%) received antenatal care at least once, with most of them going to local health centers or community health posts, where obstetricians provided care. However, even though these women were receiving antenatal care, many were not regularly tested or treated for UTIs. Our study shows that UTIs are common in pregnant women in this region. Since UTIs can lead to serious health problems for both mothers and babies, it’s important to make UTI testing a regular part of antenatal care and improve access to treatment in rural communities like San Marcos.

## Introduction

Urinary tract infections (UTIs) are typically caused by bacterial infestations of the upper and/or lower urinary tract (kidneys, ureters, bladder and urethra). UTIs are among the most common bacterial diseases in the world. In 2019, globally recorded case numbers of UTIs exceeded 404 million.^1^ Tropical Latin America had the highest age-standardised incidence of 13,853 per 100,000 population; approximately 83% of the recorded cases were women. A similar trend was observed in Andean Latin America with an incidence of 13,216 per 100,000 population or 8.4 million cases in 2019; women accounted for around 86% of all cases.^1^ UTIs are more frequent in women than men due to their shorter urethra, with an additional risk of infection escalation during pregnancy.^2^ Anatomical and functional changes of the urinary tract during pregnancy, and urine build-up from bladder compression foster favourable conditions for bacterial growth and infection.^3^ According to a recently published systematic review and meta-analysis, the global UTI prevalence in pregnant women was estimated to be 23.9%.^4^

UTIs can be classified into two subgroups: (a) symptomatic UTIs and (b) asymptomatic bacteriuria (ASB). Pregnant women with symptomatic UTIs are more likely to develop adverse pregnancy complications like miscarriage, pre-eclampsia, premature delivery and low birth weight infants compared to those with ASB.^5^ However, ASB can also affect pregnancy. Around 30% of pregnant women with untreated ASB develop acute pyelonephritis (infection of the upper urinary tract), which increases their risk of perinatal and maternal complications.^6^ While symptomatic infections can be easily detected, diagnosed and treated, asymptomatic infections often go unnoticed and require systematic screening during pregnancy for timely detection and treatment.^7^ An essential part of good antenatal care (ANC) in high-income countries includes screening for UTIs during the initial ANC visits.^8^ This practice can be quite challenging for low- and middle-income countries (LMICs) where capacity is limited.^9^ Main barriers to effective screening in LMICs include testing costs, inadequate diagnostic tools, shortage of healthcare and laboratory personnel, and limited access to services. Even when ANC is available and publicly funded, factors such as sociocultural barriers, mistrust and a lack of awareness can constrain the utilisation of ANC services and, consequently, impede the uptake of screening.^10^

According to the Peruvian National Institute of Statistics and Informatics, UTIs in pregnancy were among the most frequent gynaecological pathologies in emergency obstetric care.^11^ The Peruvian Ministry of Health recommends UTI screening through urine cultures for all pregnant women in the first trimester.^12^ In remote settings where urine cultures are unavailable, complete urine tests using urine dipsticks are recommended during the first ANC visit.^13^ Typically, only symptomatic cases undergo testing during these visits. However, in communities with low awareness of UTI symptoms, women fail to recognise their symptoms as indicative of a potential infection, and consequently do not seek medical care. Active case-finding methods can be valuable in assessing the true burden of UTIs by uncovering infections that would otherwise remain undetected. By employing such an approach at the community level, the present study aims to gather initial insights into the prevalence of UTIs among pregnant women in rural Andean communities of Peru where laboratory services are limited. Notably, there are only two other studies from Peru, both conducted in hospital settings.^14,15^ Our community-based assessment represents a novel and valuable addition to the limited knowledge of UTIs in pregnancy, particularly from resource-limited settings. Furthermore, the study explores ANC practices in the region and investigates setting-specific risk factors for UTIs. Overall, our findings serve as a foundation for developing more focused and in-depth studies on UTIs in pregnancy in the Andean region.

## Methods

### Study site and population

The study was conducted in the province of San Marcos, a high-altitude rural setting in Cajamarca, Peru, located between 1900 and 3900 m above sea level (MASL). San Marcos is one of the smaller provinces (1362 km^2^) of the 13 provinces in the Cajamarca region with a total population of 51,678 in 2020.^16^ Most of the inhabitants are farmers, living in small houses with earthen floors and adobe walls. Many families raise animals like chickens, pigs, ducks, guinea pigs, sheep and goats inside their houses, typically for consumption.^17^ Around 800 women give birth annually in the province.^18^ Since 2013, all pregnant women have access to universal health coverage from the Ministry of Health, which covers all antenatal and postnatal care.^19^ The San Marcos Province Health Network comprises three main health centres (San Marcos Health Centre (HC), Ichocan HC and Jose Sabogal HC) and 20 community-based health posts.

### Study design and participant selection

This cross-sectional study was embedded within the Peruvian Andes Multigenerational High Altitude Cohort (ALTO). The ALTO recruited all women living in the San Marcos province with an expected due date or actual delivery between February 2020 and August 2022 (index women), as well as their partners and kin (parents and grandparents).^20^ Pregnant women were identified using the pregnancy surveillance system of the local health establishments and through community visits. Subsequently, field workers conducted home visits and invited potential participants, as well as their family members (such as partners, parents and grandparents) to participate in the ALTO cohort. Individuals who intended to relocate from the province within the next 2 years were not included. Upon obtaining written consent, all participants answered a lifestyle questionnaire and attended a physical examination at baseline, and pregnant women additionally answered a maternal health questionnaire.

In parallel with the ALTO cohort enrolment, weekly lists of index women enrolled in the cohort were generated, and random visits were conducted until a subsample of 500 index women was reached. Within this subsample, fasting glucose and lipid levels as well as urine samples were collected for the investigation of non-communicable disease risk factors and proteinuria during pregnancy.^21^ These samples were collected during a second visit after baseline. The temporal separation between baseline data and sampling in our study stems from logistical constraints and the practicality of coordinating simultaneous data collection for multiple variables. However, data related to socio-demographic, household and behavioural characteristics are unlikely to undergo changes over a short period. Importantly, all data pertaining to maternal health parameters were collected on the same day as urine data sampling.

**Figure 1.**
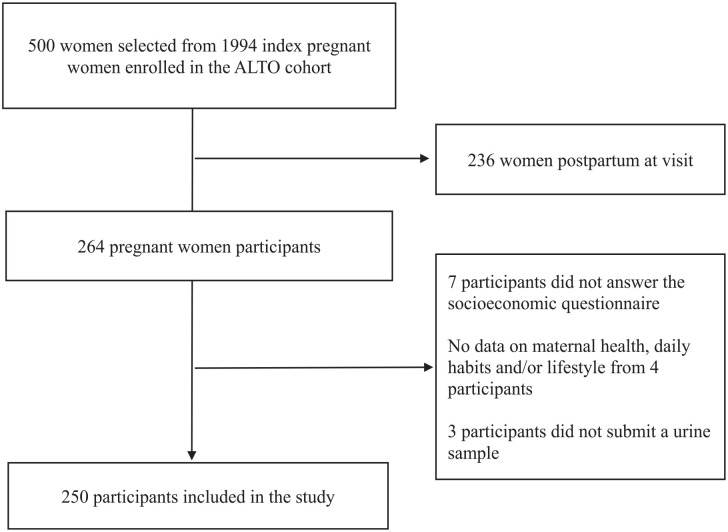
Flow diagram for the descriptive analyses of pregnant women of the San Marcos province, Cajamarca-Peru.

For this cross-sectional study, we included women of the ALTO cohort who were enrolled during pregnancy and provided a urine sample. The ALTO enrolled 1994 women, of whom 500 provided additional samples. Out of these 500 index women, 264 were still pregnant at the time of urine sample collection and were included in this study (Figure 1). By embedding our study within the ALTO, which employed active case finding and random sampling methods, we were able to obtain a representative sample for our study in San Marcos.

### Sample size calculations

Considering an estimated UTI prevalence of 20% in pregnancy,^4^ a minimum sample size of 246 pregnant women was considered sufficient to estimate the UTI prevalence with a margin error of 5% at the 95% confidence level.

### Study procedures

#### Health data, questionnaires and physical examination

For the present study, data on maternal health, daily habits and lifestyle, dietary choices, pregnancy supplements and sociodemographic characteristics of participants were obtained from the ALTO cohort baseline. All questionnaires used in the ALTO were based on standardised sources such as the Peruvian Demographic Health Survey (household characteristics and maternal health sections),^22^ WHO STEPS instrument^23^ and the International Physical Activity Questionnaire (IPAQ).^24^ A physical examination for basic health measurements including blood pressure, height, weight and haemoglobin was also conducted at baseline. Body mass index (BMI) was calculated for each participant from the data collected on height and weight during the physical examination.

#### UTI screening

Midstream urine samples were collected during the second visit. Our study emphasised comprehensive participant training to ensure precise urine sample collection to minimise the risk of contamination. Urine dipstick analyses were performed according to the manufacturers’ instructions using URIT 11V Urine Reagent Strips that contained the following analytes; ascorbic acid, specific gravity, pH, ketone, glucose, urobilinogen, bilirubin, blood, nitrite, leukocytes and proteins. For the urine examination, the strips were completely immersed in fresh urine for 2–3 s, colour changes were read against the colour chart on the container, and the results were recorded. A UTI was defined as the presence of blood (haematuria) and/or leukocyte esterase above 70 leukocytes/μL and/or nitrites in urine.^25^

### Statistical analysis

ALTO baseline data were entered using Open Data Kit and RedCap. Analyses were performed using STATA 15 Statistical software (STATA CORP, College Station, TX, USA). Descriptive statistics for a number of sociodemographic, household, behavioural, maternal health and clinical factors are presented as percentages for categorical variables and as median (Interquartile range (IQR)) for continuous variables. The UTI prevalence was estimated based on the proportion of positive UTI diagnosis from the dipstick analyses. Logistic regression was used to investigate the associations between the primary outcome UTI and nine potential risk factors. These risk factors were selected based on a comprehensive review of scientific literature,^26^ combined with expert opinions and findings from previous studies.^27^ As recommended in the Strobe reporting guidelines, results from both univariate and multivariate logistic regression models are presented. The multivariable model was pre-specified, that is, all nine identified risk factors were included without employing a variable selection procedure. This method was chosen to ensure the model incorporated all relevant variables determined by the specified criteria, avoiding the influence of data-driven decisions.

#### Study variables and measurements

For the risk-factor analysis, the primary outcome variable was a positive UTI among pregnant women, measured through urine sample testing using dipsticks, conducted during household visits at enrolment into the ALTO. The explanatory variables (risk factors) were selected from the ALTO database, using available variables collected at baseline. These include demographic variables such as age (measured in years) and education level (categorised as basic, primary, or secondary education); reproductive health variables such as gravidity (classified as first pregnancy or multiple pregnancies), history of miscarriage (recorded as yes or no), pregnancy trimester (categorised as first, second or third trimester) and abnormal glucose levels (defined as glucose levels ⩾95 mg/dL at the time of urine sample collection, recorded as yes or no); household sanitation variables such as latrine use (recorded as yes or no) and the presence of waste and animal faeces in common areas (recorded as yes or no); and socioeconomic variables such as participation in the JUNTOS program (participation in the governmental cash-transfer program recorded as yes or no). Smoking status and alcohol consumption were not included in the analysis because prior research indicated low exposure levels in this population.^27^ Additionally, our data also confirmed that most of our participants were non-smokers.

## Results

### Study participants

Of the 264 women who were still pregnant during urine sampling, 14 were excluded from analyses due to missing information. Data from 250 participants were analysed (Figure 1). During urine sampling, 6% of the participants were in their first trimester of pregnancy, 46% in their second and 48% in their third trimester (Table 1). Time periods between baseline and urine sampling varied across participants with a median of 4 weeks (IQR: 1–7).

**Table 1.** Sociodemographic, household and behavioural characteristics of pregnant women of the San Marcos province, Cajamarca-Peru.

Characteristics	Total	Positive UTI	Negative UTI
	*n* = 250	*n* = 69	*n* = 181
**A. Sociodemographic characteristics**			
Age (in years)*	28 (22–33)	28 (21–33)	28 (22–34)
Gestational age (in weeks)*	26 (20–33)	27 (20–33)	26 (20–33)
Number of participants in each trimester*			
First trimester (0–13 weeks)	15 (6%)	1 (1%)	14 (8%)
Second trimester (14–26 weeks)	116 (46%)	32 (47%)	84 (46%)
Third trimester (27–40 weeks)	119 (48%)	36 (52%)	83 (46%)
Married or cohabitant	230 (92%)	65 (94%)	165 (91%)
Incomplete primary schooling	40 (16%)	11 (16%)	29 (16%)
Altitude of living (MASL)			
<2500	123 (50%)	35 (51%)	88 (49%)
2500–3000	88 (35%)	26 (38%)	62 (34%)
>3000	39 (15%)	8 (11%)	31 (17%)
Occupation			
Unemployed/housewife	211 (84%)	55 (80%)	156 (86%)
Raising animals^a^	14 (6%)	6 (9%)	8 (4%)
Student	17 (7%)	4 (6%)	13 (7%)
Others	8 (3%)	4 (5%)	4 (3%)
SIS health insurance	246 (98%)	68 (99%)	178 (98%)
Cash transfer programme ‘JUNTOS’	79 (32%)	19 (28%)	60 (33%)
**B. Household characteristics**			
Adobe wall	178 (71%)	48 (70%)	130 (72%)
Roof with tiles	158 (63%)	44 (64%)	114 (63%)
Earthen floor	150 (60%)	34 (49%)	116 (64%)
House owner	192 (77%)	48 (70%)	144 (80%)
Piped water	234 (94%)	65 (94%)	169 (93%)
Treated drinking water	169 (68%)	47 (68%)	122 (67%)
Toilet type			
House with sewage system connection	90 (36%)	28 (41%)	62 (34%)
House with latrine	158 (63%)	39 (57%)	119 (66%)
Open defecation	2 (1%)	2 (2%)	0 (%)
Overcrowded house (>3 people sleeping per room)	10 (4%)	1 (2%)	9 (5%)
Trash observed	216 (86%)	57 (83%)	159 (88%)
Animal faeces observed	212 (85%)	54 (78%)	158 (87%)
**C. Behavioural characteristics**			
Daily fruit intake	144 (58%)	41 (59%)	103 (57%)
Daily vegetable intake	85 (34%)	26 (38%)	59 (33%)
Physical activity			
Low	50 (20%)	16 (23%)	34 (19%)
Moderate	163 (65%)	41 (60%)	122 (67%)
High	37 (15%)	12 (17%)	25 (14%)
Handwashing after toilet use	242 (97%)	68 (99%)	174 (96%)

Continuous variables are presented as median (IQR), and categorical variables are presented as absolute frequency (percentages). SIS: Sistema Integral de Salud; MASL: metres above sea level.

a For sale and consumption.

* Data collected during urine sample collection. Please note that categorical variables are presented as column percentages (rounded to the nearest whole number) and only those variables for which all categories are presented will equate to 100%.

#### Prevalence of UTI

In the screening of urine samples from our pregnancy cohort (*n* = 250), a total of 69 participants tested positive for a UTI, corresponding to a prevalence of 27.6% (CI: 22.4%–33.5%). Upon stratifying by trimester, the prevalences were 6.6%, 27.5% (CI: 20.1%–36.4%) and 30.2% (CI: 22.6%–39.1%) for pregnant women in the first, second and third trimesters, respectively. We did not calculate a confidence interval (CI) for the first-trimester group due to the small sample size (*n* = 15).

#### Sociodemographic characteristics

The study participants had an overall median age of 28 years (IQR: 22–33), which was similar in the UTI-positive (Group 1) and UTI-negative groups (Group 2) (Table 1). The median gestational age during sample collection was 26 weeks (IQR: 20–33). Most of the participants were married or cohabiting and had completed primary education. Around 84% of participants were housewives, 6% raised animals for sale and consumption and 7% were students. Some 3% had other professions as teachers, pharmacists and police officers, among others. Almost everyone (98%) was affiliated with SIS (Seguro Integral de Salud), a national health insurance system for all Peruvians, especially those living in poverty or extreme poverty.^28^ The SIS scheme provides free health care at all public health centres for pregnant mothers. About 32% were registered in the governmental cash transfer programme JUNTOS, which provides monetary incentives to Peruvian households in poverty and extreme poverty.^29^ Most of the sociodemographic characteristics were similar in both groups (Table 1).

#### Household characteristics

Most of our participants lived in houses with adobe walls, roof tiles and earthen floors, and 192 (77%) of them said they were the house owners (Table 1). A total of 10 (4%) participants lived in overcrowded conditions, with more than three people sleeping per room.^30^ Animal faeces and trash were found in the courtyards and common areas. Some 169 (68%) confirmed they treated water before drinking it. Only 1% of households in our study practiced open defecation (i.e. river, ditch, canal, bush and field), the remainder was connected to the public sewage system (36%) or had latrines (63%). Furthermore, 94% of the households had piped water connections.

#### Behavioural characteristics

Self-reported behavioural characteristics of our study participants are presented in Table 1. In both groups, half of the participants reported a daily fruit-rich diet, while less than half reported a daily vegetable intake. A majority of the participants had an active lifestyle based on the IPAQ scores and almost everyone reported appropriate handwashing behaviour after using the toilet.

#### Maternal health during pregnancy

Clinical parameters measured at sample collection are presented in Table 2. Similar values for median total cholesterol, high-density lipoprotein (HDL) and triglyceride levels were observed in both UTI-positive and UTI-negative groups. A slightly higher percentage of pregnant women in the UTI-positive group (23% vs. 17%) had elevated glucose levels (⩾95 mg/dL). The above clinical parameters, except glucose and HDL, increased with time of gestation, peaking in the third trimester of pregnancy (Table S1). A median systolic blood pressure of 98 mmHg and diastolic blood pressure of 66 mmHg were observed in our sample.^31^ Both were consistent across both UTI-positive and UTI-negative groups. In total, 34% of the participants had a normal BMI; 45% were overweight and 21% were obese (Table 2). History of gastritis, preeclampsia and gestational diabetes was low (6%, 2% and 0%, respectively) in our participants. None of the participants reported an intake of antimicrobials in the past 3 months.

**Table 2.** Maternal health of pregnant women of the San Marcos province, Cajamarca-Peru.

Characteristics	Total *n* = 250	Positive UTI *n* = 69	Negative UTI *n* = 181
Cholesterol (mg/dL)*	189 (151–223)	189 (160–228)	187 (149–220)
HDL (mg/dL)*	44 (37–52)	45 (38–53)	44 (37–51)
Triglyceride (mg/dL)*	200 (151–275)	206 (152–273)	199 (151–276)
Glucose ⩾ 95 mg/dL	47 (19%)	16 (23%)	31 (17%)
Systolic blood pressure (mmHg)*	98 (92–105)	96 (90–106)	99 (92–105)
Diastolic blood pressure (mmHg)*	66 (61–71)	63 (58–68)	67 (62–71)
Blood pressure >140/90 mmHg*^31^	4 (2%)	3 (4%)	1 (1%)
BMI (kg/m^2^)	26 (24–29)	27 (24–29)	26 (24–29)
BMI categories (kg/m^2^)			
Normal (18.5–24.9)	86 (34%)	21 (30%)	65 (36%)
Overweight (25.0–29.9)	113 (45%)	33 (48%)	80 (44%)
Obesity (⩾30)	51 (21%)	15 (22%)	36 (20%)
History of gastritis	14 (6%)	4 (6%)	10 (6%)
History of preeclampsia	4 (2%)	1 (1%)	3 (2%)
History of gestational diabetes	1 (0%)	1 (1%)	0 (0%)
At least one ANC visit			
Yes	241 (96%)	69 (100%)	172 (95%)
No	5 (2%)	0 (0%)	5 (3%)
First ANC not received	4 (2%)	0 (0%)	4 (2%)
Gestational age at first ANC visit (weeks)	10 (7–15)	11 (8–16)	10 (7–14)
Vitamin supplements (missing: 9)	223 (89%)	62 (90%)	161 (89%)
Iron supplements (missing: 9)	222 (89%)	64 (93%)	158 (87%)
First pregnancy	76 (30%)	18 (26%)	58 (32%)
History of miscarriage	38 (15%)	11 (16%)	27 (15%)

Continuous variables are presented as median (Interquartile range), and categorical variables are presented as absolute frequency (percentages). ANC: antenatal care; BMI: body mass index; HDL: high-density lipoprotein.

* Data collected during urine sample collection. Please note that categorical variables are presented as column percentages (rounded to the nearest whole number) and only those variables for which all categories are presented will equate to 100%. Any instances of missing values are explicitly noted alongside the respective variable.

We observed a high attendance at ANC visits, 96% of the participants reported having received ANC at least once (Table 2). Nearly half of those who had previously given birth (*n* = 174) reported having attended a median of nine ANC visits during their last pregnancy (Table S2). Furthermore, upon reviewing clinical histories from the health centre, 62% of our study participants had undergone at least one complete urine test during their ANC visits; 72% of them had one test performed during their first visit. In all cases, the urine tests were performed using urinary dipsticks; only one case involved a urine culture (data not shown). It should be noted that the specific purpose of these tests was unclear as the available data from the health centre did not provide information on the diagnosis of UTIs.

#### Risk factors for UTI

Women with a history of previous pregnancies or miscarriages and for whom we had measured abnormal glucose levels had higher odds of UTI with odds ratios (OR) of 1.13 (CI: 0.50–2.60) and 1.35 (CI: 0.64–2.81), respectively, in the multivariable analysis. Women who had completed secondary schooling had increased odds of UTI (OR: 1.35; CI: 0.53–3.42), while those who participated in the cash transfer programme had decreased odds (OR: 0.75; CI: 0.40–1.44). Increased odds for a UTI were also observed in women from households where waste was observed indoors (OR: 1.71; CI: 0.76–3.87). However, none of these associations were statistically significant in both univariable and multivariable analyses (Table 3).

**Table 3.** Univariable and multivariable analyses of determinants of UTI in pregnant women of the San Marcos province, Cajamarca-Peru.

Risk factors	Univariable analysis			Multivariable analysis		
	OR	95% CI	*p*-Value	aOR	95% CI	*p*-Value
Age (years)	0.99	0.96–1.03	0.82	0.97	0.92–1.02	0.20
Education						
Basic	1.00			1.00		
Primary schooling	0.80	0.35–1.84	0.60	0.80	0.32–2.03	0.64
Secondary schooling	1.24	0.55–2.79	0.60	1.35	0.53–3.42	0.53
Participation in a cash transfer programme ‘JUNTOS’	0.77	0.42–1.41	0.40	0.75	0.40–1.44	0.39
Gravidity > 1	1.34	0.72–2.49	0.36	1.90	0.84–4.30	0.12
History of miscarriage	1.08	0.51–2.32	0.84	1.13	0.50–2.60	0.77
Pregnancy trimester						
First	1.00			1.00		
Second	5.33	0.67–42.23	0.11	5.42	0.67–43.87	0.11
Third	6.08	0.77–47.92	0.09	5.93	0.73–47.91	0.10
Latrine use	0.68	0.38–1.19	0.17	0.69	0.38–1.25	0.22
Abnormal glucose levels	1.46	0.74–2.88	0.28	1.35	0.64–2.81	0.43
Waste observed inside the house	1.52	0.71–3.27	0.28	1.71	0.76–3.87	0.14

OR: odd ratio; aOR: adjusted OR; CI: confidence intervals.

## Discussion

The present study aimed to provide a baseline understanding of the prevalence of UTIs among pregnant women in a rural Peruvian Andean province. A community-based approach was applied to screen for UTIs in a subset of a population-based cohort (ALTO). To our knowledge, this is the first published report on UTI prevalence in a rural high-altitude setting in the Andes.

In a recent systematic review across nine Latin American countries, pooled prevalence estimates for ASB, lower UTI and pyelonephritis in pregnant women were 18.4%, 7.5% and 2.3%, respectively.^32^ The UTI prevalence of 27.6% found in our setting exceeds these individual rates. However, in the systematic review, there is substantial heterogeneity in the pooled estimates, ranging from a prevalence of 1.7% in Mexico to 56% in Brazil. Within Peru, the two studies of Quiroz-del Castillo and Apolaya-Segura^14^ and Villamonte et al.,^15^ reported prevalence rates of 7.4% and 17.7%, with the latter specifically representing the prevalence of ASB. This variation underscores the importance of considering regional differences in UTI prevalence and its assessment when interpreting and comparing study findings.

In rural areas like San Marcos, limited access to proper hygiene facilities (e.g. problems with sewer lines and waste disposal) elevates UTI risks. Additionally, constrained treatment accessibility may lead to prolonged UTI episodes, increasing prevalence and introducing risks such as intrauterine growth restriction (IUGR), low birth weight and preterm delivery among others. Notably, a substantial number of our participants were in their second (46%) and third trimesters (48%) of pregnancy, potentially amplifying the risk of UTI amidst various physiological changes.^2,3^ While the association between later stages of pregnancy and an increased risk of UTIs is not fully established,^33^ it is possible that it contributes to the observed prevalence in our study. Additionally, our systematic screening strategy involving household visits, not only allowed for the identification of asymptomatic cases but also identified individuals who were symptomatic but unaware of their symptoms. This active case-finding approach, especially in settings where routine UTI screening is not implemented, results in a higher reported prevalence compared to passive case-finding at healthcare facilities. It should be noted that the reported increase in prevalence through active case-finding is influenced by various factors, including the screening method used, coverage and intensity of screening efforts, as well as the characteristics of the population under study, and therefore, must be interpreted with caution.^34^

Throughout pregnancy, multiple factors increase the likelihood of developing UTIs. Well-known risk factors include age, gestational age, sexual activity, multigravidity, multiparity, history of UTI, low socioeconomic status and gestational diabetes.^26^ Nonetheless, existing studies have not consistently identified significant associations with UTIs as risk factors often vary across different study settings.^35,36^ Through our research, we aimed to investigate risk factors for UTIs specific to a resource-constrained Andean setting. We examined factors such as the presence of animal faeces in the house and latrine use. There is evidence that domestic pets can harbour and spread pathogenic bacteria, such as *Escherichia coli* responsible for UTIs among dog owners.^37^ Given that many households in our study reared animals such as chickens, pigs, ducks, guinea pigs, sheep and goats indoors, we chose to investigate whether the presence of animal faeces and waste in common areas is linked to an increased risk of UTIs. This variable was also chosen to serve as an indicator of suboptimal hygiene practices. Further, we assessed latrine use to determine if there was an association between open defecation practices and UTIs. Despite these efforts, our analysis did not reveal any statistically significant associations, possibly due to a small sample size. This also limited our ability to stratify the analysis by age, gestational age and gravidity. The short duration of our study may have also impeded capturing the dynamics of risk factor exposures and their impact on outcomes. Our study is exploratory in nature. Studies with larger sample sizes and longer duration are needed to better describe UTI-specific risk factors, providing stronger evidence for effective setting-specific prevention and management of UTIs during pregnancy. Moreover, the inclusion of a control group in future endeavours is needed to explore factors associated with pregnancy that may contribute to increased UTI risk.

Attendance at ANC visits in our setting was notably high, consistent with the Peruvian Demographic Health Survey, 2020.^22^ The survey reported that 93.9% of women received professional ANC in 2020, with over 90% of women attending six or more ANC visits. ANC is recommended during pregnancy to ensure a positive pregnancy experience and optimal pregnancy outcomes.^38^ While the World Health Organization recommends a minimum of eight ANC visits, the Peruvian national guideline recommends at least six.^39^ Peru offers free ANC through initiatives like SIS and JUNTOS. Our findings indicate that 96% of our participants received ANC at least once, with a mean gestational age of 10 weeks at the first visit. This highlights both the accessibility to care and the positive care-seeking behaviour in the region. However, only about 45% of all our study participants underwent UTI screening during their first ANC visit (based on health centre data), indicating potential inconsistency with national guidelines. Despite robust ANC attendance, the absence of UTI screening and treatment poses a potential risk for UTI-related complications for both, mother and child. This gap in care can lead to adverse outcomes and addressing it could substantially contribute to improving maternal and foetal health outcomes. Further, as this Andean population has already shown strong local cultural perceptions regarding heart diseases,^40^ it is likely that an understanding of UTIs in the population also has a cultural influence. Qualitative research on women’s understanding and perceptions of UTIs, personal hygiene and reproductive health practices could provide the insights needed to improve antenatal and postnatal services, and to design interventions and educational programmes aimed at reducing UTIs among pregnant women.

### Limitations

Our cross-sectional design assessed UTI prevalence at a single time point and did not account for multiple episodes or distinguish between different infection types (e.g. acute, unresolved, asymptomatic or recurrent infections). Secondly, the prevalence estimate relied solely on urine dipstick analyses, susceptible to false positives due to contamination, dietary interference or inherent dipstick limitations. While gold standard tools like urine cultures with clinical evaluations are recommended, limited resources prevented their use in our study. Nonetheless, a separate study involving urine cultures on a sub-sample of our study population corroborated our dipstick-based findings and identified *Escherichia coli, Klebsiella* sp., *Staphylococcus* sp. and *Candida albicans* as the causative agents of UTIs. The present study was embedded within a cohort that was not designed to study the epidemiology of UTIs. Consequently, we were unable to administer a questionnaire tailored specifically to UTIs, resulting in the absence of data on known UTI risk factors such as previous UTI history, urinary habits, sexual activity during pregnancy, contraceptive use, personal hygiene and UTI-specific symptoms. It is crucial to recognise the potential influence of recall bias in our study. This draws from utilising standardised questions in the ALTO mother study (our source data), which may have affected the collection of demographic and socioeconomic data (Tables S3). Conversely, we emphasise the reliability of maternal health information which was derived from women’s health cards. Lastly, our study was conducted within the specific context of San Marcos in Peru, and the sample characteristics reflect the unique demographic and healthcare landscape of San Marcos. Therefore, our findings may have limitations in generalisability to other populations but can be extended to rural Andean Peru communities with similar characteristics.

This is the first study exploring UTIs among pregnant women in the San Marcos province of rural Andean Peru, an area with approximately 800 annual births.^18^ Our study encompassed a significant one-third of this population, thus providing valuable and solid insights into this specific context. During pregnancy, asymptomatic UTIs are common and often go unnoticed without screening. We screened for UTIs at the household level, unlike other studies that included participants attending ANC in the clinics and health centres. This approach enables a more accurate estimation of UTI prevalence as it is independent of a woman’s access to and attendance of ANC. Lastly, our study offers a comprehensive analysis of the characteristics of pregnant women in our region, which is seldom obtained from studies conducted solely in clinical settings.

## Conclusions

Our household-based study conducted in the high-altitude setting of San Marcos, Andean Peru revealed a UTI prevalence of 27.6% among pregnant women. The clinical implications of our research emphasise the need for integrating UTI screening and management into routine ANC services. This integration becomes especially crucial given the observed high ANC attendance, indicating a strong foundation for healthcare engagement in the community. The findings advocate for enhanced screening measures to ensure the comprehensive well-being of pregnant women and their infants. Moreover, it is essential to educate pregnant women about common UTI symptoms and urge them to seek medical attention if they experience any UTI-specific symptoms. Raising awareness among pregnant women and their families about the consequences of UTIs during pregnancy and the advantages of timely treatment could enhance the uptake of screening programmes and treatment.

## Supplemental Material

sj-docx-1-whe-10.1177_17455057241294215 – Supplemental material for Prevalence of urinary tract infections in pregnancy in rural Andean communities of Peru Supplemental material, sj-docx-1-whe-10.1177_17455057241294215 for Prevalence of urinary tract infections in pregnancy in rural Andean communities of Peru by Apoorva Venkatesh, Giuliana Sanchez-Samaniego, Daniel Mäusezahl, Jan Hattendorf and Stella M Hartinger in Women's Health

sj-docx-2-whe-10.1177_17455057241294215 – Supplemental material for Prevalence of urinary tract infections in pregnancy in rural Andean communities of Peru Supplemental material, sj-docx-2-whe-10.1177_17455057241294215 for Prevalence of urinary tract infections in pregnancy in rural Andean communities of Peru by Apoorva Venkatesh, Giuliana Sanchez-Samaniego, Daniel Mäusezahl, Jan Hattendorf and Stella M Hartinger in Women's Health

## Declarations

**Ethical approval and consent to participate:** The ALTO cohort and the cross-sectional study were approved by the Universidad Peruana Cayetano Heredia (UPCH) Ethical Review Board (No. 740-26-19 and No. 356-01-21) and by the Ethikkommission Nordwest- und Zentralschweiz (Res_2020-00088 and AO_2020-00039). The projects were conducted in accordance with the Declaration of Helsinki for biomedical research involving human subjects. Participants with a positive UTI diagnosis were referred to the health centre. All participants provided written consent to participate in the ALTO cohort and to provide urine samples; those who did not consent were excluded.

**Consent for publication:** Not applicable.

**Availability of data and materials:** The data that support the findings of this study are available from the corresponding author upon reasonable request.
