# Supplementary material for: Prevalence of urinary tract infections in pregnancy in rural Andean communities of Peru
Source: Womens Health (Lond). 2024 Nov 5;20:17455057241294215. doi: 10.1177/17455057241294215 (PMC11536492; doi:10.1177/17455057241294215)
Supplement: sj-docx-2-whe-10.1177_17455057241294215 – Supplemental material for Prevalence of urinary tract infections in pregnancy in rural Andean communities of Peru [file sj-docx-2-whe-10.1177_17455057241294215.docx]

| Table S1. Median values for clinical parameters stratified by gestation period | | | |
| --- | --- | --- | --- |
| **Clinical parameters** | **First trimester** | **Second trimester** | **Third trimester** |
|  | (0-13 weeks) | (14-26 weeks) | (27-40 weeks) |
|  |  |  |  |
| Glucose (mg/dL) | 83 [79-93] | 82 [74-92] | 80 [68-91] |
| Total cholesterol (mg/dL) | 151 [119-173] | 175 [137-209] | 208 [169-237] |
| HDL (mg/dL) | 38 [32-49] | 44 [36-53] | 44 [38-52] |
| Triglyceride (mg/dL) | 115 [95-157] | 183 [141-226] | 246 [184-311] |
| Continuous variables are presented as median [Interquartile range] | | | |

**Supplementary information**

| Table S2. Details of last pregnancy for 174 participants who were previously pregnant | |
| --- | --- |
|  | **Last pregnancy** |
|  | **n=174** |
| **Number of previous pregnancies** |  |
| 1 | 71 (41%) |
| 2 | 56 (32%) |
| 3 | 30 (17%) |
| ≥4 | 17 (10%) |
| **Twins or triplets** | |
| No | 173 (99%) |
| Yes | 1 (1%) |
| **Received ANC** |  |
| No | 13 (7%) |
| Yes | 161 (93%) |
| **Number of ANC visits** (Missing:13) | 9 [7-10] |
| **Care provider** (Missing:13) | |
| Doctor/Physician | 28 (16%) |
| Obstetrician | 143 (82%) |
| Nurse | 51 (29%) |
| One of the above | 112 (64.3%) |
| Two of the above | 36 (24.9%) |
| All three | 13 (7.4%) |
| **ANC location** |  |
| Local health post | 67 (39%) |
| Health centre | 87 (50%) |
| Hospital | 10 (6%) |
| Pharmacy | 0 (0%) |
| Clinic or private hospital | 2 (1%) |
| Private consultant | 1 (1%) |
| At home | 0 (0%) |
| Others | 4 (2%) |
| **Place of delivery** | |
| Local health post | 22 (13%) |
| Health centre | 67 (39%) |
| Hospital | 50 (29%) |
| Private hospital | 4 (2%) |
| At home | 19 (11%) |
| Road | 2 (1%) |
| Continuous variables are presented as median [Interquartile range] and categorical variables are presented as absolute frequency (percentages) | |

| Table S3. Close-ended questions from the ALTO questionnaire presented in Tables 1 and 2 | |
| --- | --- |
| Question (Translated) | Options |
| Table 1 | |
| What was the last GRADE you completed this year? |  |
|  | 00 = None |
|  | Grade = 01-11 |
|  | 12=Cunamas |
|  | 13= Sup. No Univ. Incomplete |
|  | 14 = Sup. No Univ. Complete |
|  | 15=Univ. completed |
|  | 16=Univ. uncomplete |
|  | 18= Kindergarden /childcare /PRONOI |
|  | 19= EB Inicial (3-5 years old) |
|  | 66= Other (Specify) |
|  | 77 = NS |
| Civil status |  |
|  | 01= Partner |
|  | 02= Separated |
|  | 03= Married |
|  | 04= Widow |
|  | 05= Divorced |
|  | 06=Single |
|  | 77= DK |
|  | 88= NA |
| Economic sector code of the work/occupation (free text-coded) | Refer Table 2A for codes |
| Is he/she registered in a health insurance programme? |  |
|  | 00=Not affiliated |
|  | 01=ESSALUD/Before IPSS |
|  | 02=Military or Police |
|  | 03=SIS |
|  | 04=EPS |
|  | 05=Private Health Insurance |
|  | 66=Other (Specify) |
|  | 77 = DK |
|  | 88=NA |
|  |  |
| Wall (Interviewer: observe and, if necessary, ask) |  |
|  | 11 - Ladrillos bloque de cemento |
|  | 12 - Piedra o sillar con cal o cemento |
|  | 13 - Adobe o tapia tarrajeada |
|  | 21 - Adobe o tapia sin tarrajear |
|  | 22 - Tablones/Madera |
|  | 23 - Quincha/Caña con barro |
|  | 24 - Piedra con barro |
|  | 31 - Caña/Bambu/Piedra/Palma/Tronco/Tabique |
|  | 32 - Estera |
|  | 33 - Carton |
|  | 34 - Triplay |
|  | 35 - Sin paredes |
|  | 96 - Otros |
|  | 77 - NS |
| Roof (Interviewer: observe, if necessary, ask) |  |
|  | 11 - Concreto armado |
|  | 12 - Tejas |
|  | 21 - Plancha de calamina/Fibra de cemento o similares |
|  | 22 - Madera |
|  | 23 - Caña o estera con torta de barro |
|  | 31 - Paja, hojas de palma etc |
|  | 32 - Estera |
|  | 33 - Carton |
|  | 34 - Sin techo |
|  | 96 - Otros |
|  | 77 - NS |
| Floor (Interviewer: observe, if necessary, ask) |  |
|  | 11 - Parquet o madera pulida |
|  | 12 - Lamina asfaltica, vinilico o similar |
|  | 13 - Loseta, terrazos o similares |
|  | 21 - Mader entablados |
|  | 22 - Pona |
|  | 91 - Tierra o arena |
|  | 96 - Otros |
|  | 77 - NS |
| Is a member of your household the owner of the house where you live? | 00=No, 01=si, 77=DK |
| Do you treat the water that the family consumes? | 00=No, 01=si, 77=DK |
| What kind of toilet facility do members of your household usually use? |  |
|  | 11 - Conectado a red publica de desague dentro de la casa |
|  | 12 - Conectado a la red publica de desague, fuera de la vivienda pero dentro del edificio |
|  | 21 - Pozo septico / Tanque septico |
|  | 31 - Letrina, mejorada ventilada |
|  | 32 - Letrina mejorada ecologica, abonera, compostera |
|  | 33 - Letrina mejorada, colgante flotante |
|  | 34 - Pozo ciego o negro con tratamiento de cal, estiercol , aserrin, arena |
|  | 35 - Pozo ciego o negro |
|  | 41 - Rio, acequia, canal |
|  | 51 - No hay servicio (matorral, campo) |
|  | 66 - Otro |
| In the courtyard / common area of the household - Do you observe the following: waste(food and paper) on the floor | 00= No, 01=Yes, 66= was not observed, 77 = DK |
| In the courtyard / common area of the household - Do you observe the following: animal faeces on the floor | 00= No, 01=Yes, 66= was not observed, 77 = DK |
| Daily fruit intake | categorical (low=1, moderate=2, high=3) |
| Daily vegetable intake | binary (yes=0, no=0) |
| Physical activity | binary (yes=0, no=0) |
| In what moments do you wash your hands? Select any that apply: After using the toilet | 00=No, 01=Yes, 88=NA |
| **Table 2** | |
| Did a doctor ever tell you that you have one of the following:Gastritis | 1=yes 2=no |
| Did a doctor ever tell you that you have one of the following:Preclampsia | 1=yes 2=no |
| Did a doctor ever tell you that you have one of the following:Gestational diabetes | 1=yes 2=no |
| Do you receive prenatal care during your pregnancy? | 1=yes 2=no |
| Are you receiving vitamin? | yes=01, no=00 |
| Are you receiving iron supplements? | yes=01, no=00 |

| **TABLE # 2A – OCCUPATION** | | |
| --- | --- | --- |
| **Linked to agriculture/fishery/forestry** | **Not linked to agriculture/fishery/forestry** | **Unemployed/non-remunerated** |
| 01 = farmer (land owner) | 09 = self-employed, small enterprise | 17 = Unemployed |
| 02 = farmer with salary | 10 = self-employed, services | 18 = Housewife |
| 03 = laborer | 11 = employee with salary | 19 = Dependent (children or) |
| 04 = farmer (animals) | 12 = occasional employee | 20 **=** Other, non-remunerated (specify) |
| 05 = Forestal | 13 = craftsman | 77 = NS 88 = NA |
| 06 = Fisherman | 14 = merchant |  |
| 07 = works for the family, non-remunerated | 15 = works for the family, non-remunerated |  |
| 08 = Other (linked with agriculture) | 16 = Other ( not linked with agriculture) |  |

| Table S4. Close-ended questions from the ALTO questionnaire presented in Table S2 | |
| --- | --- |
| **Questions (Translated)** | **Options** |
| Have you had twins or triplets before? | yes=01, no=00, NS=77 NA=88 |
| During your previous pregnancy, have you received prenatal care during your pregnancy? | yes=01, no=00, NS=77 NA=88 |
| During your previous pregnancy, Who evaluated you during your prenatal care? General Practitioner | yes=01, no=00 |
| During your previous pregnancy, Who evaluated you during your prenatal care? Obstetrician | yes=01, no=00 |
| During your previous pregnancy, Who evaluated you during your prenatal care? Nurse ( technician) | yes=01, no=00 |
| During your previous pregnancy, Who evaluated you during your prenatal care? Health promoter | yes=01, no=00 |
| During your previous pregnancy, Who evaluated you during your prenatal care? Midwife | yes=01, no=00 |
| During your previous pregnancy, Who evaluated you during your prenatal care? DK | yes=01, no=00 |
| During your previous pregnancy, Who evaluated you during your prenatal care? Other | yes=01, no=00 |
| During your previous pregnancy, Who evaluated you during your prenatal care? Other |  |
| During your previous pregnancy, Where did you received your prenatal care? (3 options) Health Post | yes=01, no=00 |
| During your previous pregnancy, Where did you received your prenatal care? (3 options) Health Centre | yes=01, no=00 |
| During your previous pregnancy, Where did you received your prenatal care? (3 options)Hospital | yes=01, no=00 |
| During your previous pregnancy, Where did you received your prenatal care? (3 options)Private Hospital | yes=01, no=00 |
| During your previous pregnancy, Where did you received your prenatal care? (3 options)Drugstore | yes=01, no=00 |
| During your previous pregnancy, Where did you received your prenatal care? (3 options) Private Doctor | yes=01, no=00 |
| During your previous pregnancy, Where did you received your prenatal care? (3 options) At Home | yes=01, no=00 |
| During your previous pregnancy, Where did you received your prenatal care? (3 options)Other | yes=01, no=00 |
| During your previous pregnancy, Where did you received your prenatal care? (3 options)Other |  |
| In your previous pregnancy, where did you give birth in your last pregnancy? | 01=Health Post |
|  | 02=Health Centre |
|  | 03=Hospital |
|  | 04=Private Hospital |
|  | 05=Drugstore |
|  | 06= Private doctor |
|  | 07=at home |
|  | 08= on the way |
|  | 09= Other |
